# Demodex Infestation in Patients With Atopic Dermatitis Treated With Dupilumab: A Case–Control Study

**DOI:** 10.1111/1346-8138.70372

**Published:** 2026-06-28

**Authors:** Pinar Dursun, Ayse Nur Saribas Yildirim, Ayca Yazici

**Affiliations:** ^1^ Department of Dermatology Mersin University Faculty of Medicine Mersin Turkiye

**Keywords:** atopic dermatitis, Demodex, dupilumab, facial dermatoses

## Abstract

Atopic dermatitis (AD) is a chronic inflammatory skin disease in which dupilumab, an interleukin‐4 receptor alpha antagonist, has become a cornerstone therapy for moderate‐to‐severe disease. However, persistent facial and neck erythema and related symptoms have been increasingly reported during dupilumab treatment, and the underlying mechanisms remain incompletely understood. Demodex species, common components of the cutaneous microbiota, have been proposed as potential contributing factors. This retrospective case–control study evaluated Demodex infestation in patients with moderate‐to‐severe AD receiving dupilumab therapy compared with AD patients not receiving systemic immunosuppressive treatment. A total of 61 patients were included: 31 dupilumab‐treated patients and 30 controls. Demodex density was assessed using standardized skin surface biopsy, with ≥ 5 mites/cm^2^ considered positive infestation. Clinical features, laboratory parameters, and treatment characteristics were analyzed. Demodex positivity was significantly more frequent in dupilumab‐treated patients than in controls (45.2% vs. 6.7%, *p* = 0.001). All dupilumab‐treated patients had achieved at least a 75% improvement in Eczema Area and Severity Index at the time of assessment. Within the dupilumab group, Demodex‐positive patients were older than Demodex‐negative patients, while no significant differences were observed regarding sex, eosinophil counts, or total IgE levels. Most Demodex‐positive patients reported persistent facial erythema and pruritus, often accompanied by ocular symptoms. Although a causal relationship cannot be established due to the retrospective design, these findings suggest that Demodex infestation may coexist with persistent head‐and‐neck symptoms in AD patients receiving dupilumab. Evaluation for Demodex infestation may be considered clinically relevant in patients presenting with atypical or treatment‐resistant facial involvement during dupilumab therapy.

## 1 Introduction

Atopic dermatitis (AD) is a chronic, relapsing, non‐infectious inflammatory dermatosis characterized by intense pruritus. Although it is more commonly observed in the pediatric population, the lifetime prevalence of AD may reach up to 20% [1]. In adults, the prevalence of AD ranges between 3% and 10%, with approximately 20% of cases presenting in moderate‐to‐severe forms [2, 3].

The etiopathogenesis of AD is multifactorial and involves epidermal barrier dysfunction, immune dysregulation, genetic predisposition, and environmental factors [4]. Interleukin‐4 (IL‐4) and IL‐13, predominantly produced by T helper 2 (Th2) cells, play central roles in disease pathogenesis by promoting type 2 inflammation [5].

Dupilumab is the first biologic agent approved for the treatment of moderate‐to‐severe AD. By binding to the IL‐4 receptor alpha subunit, it inhibits both IL‐4 and IL‐13 signaling pathways, resulting in downregulation of Th2‐mediated inflammation [6]. The most commonly reported adverse events include injection‐site reactions, nasopharyngitis, nausea, and headache [7]. Other less frequent adverse events, such as dry eye, conjunctivitis, blepharitis, head and neck erythema or dermatitis, exacerbation of cutaneous T‐cell lymphoma, alopecia areata, and arthritis, have also been reported [7].

Among these adverse events, persistent facial and neck erythema has attracted increasing attention, leading to investigation of potential contributing factors, including Demodex spp. infestation [8]. To date, the available evidence regarding Demodex‐associated eruptions during dupilumab therapy has been limited to case reports and small case series, and controlled comparative data remain scarce. *Demodex folliculorum* (*D. folliculorum*) and *Demodex brevis* ( *D. brevis* ) are ectoparasites that constitute part of the normal human skin microbiota. *D. folliculorum* resides primarily within the follicular infundibulum, whereas *D. brevis* inhabits deeper sebaceous glands [9]. *D. folliculorum* is more prevalent and is typically found in greater numbers per follicle. Although Demodex mites are commonly detected at low densities in healthy individuals (≤ 5 mites/cm^2^), colonization rates increase with age and approach near universality in individuals older than 70 years [10, 11].

Demodex proliferation may be facilitated by several factors, including immunosuppression, diabetes mellitus, vasodilation, and sebaceous gland hyperplasia [12]. Clinically, Demodex infestation has been associated with conditions such as blepharitis, rosacea, meibomian gland dysfunction, demodicosis, and seborrheic dermatitis [13]. Nevertheless, its pathogenic significance remains controversial, given the high prevalence of asymptomatic colonization and the inconsistent correlation between mite density and clinical severity [8].

Chen et al. classified demodicosis into primary and secondary forms [14]. Primary demodicosis occurs in the absence of an underlying dermatosis, whereas secondary demodicosis develops in association with other skin diseases or immunomodulatory conditions. Alternatively, Forton et al. proposed a classification distinguishing inflammatory and non‐inflammatory demodicosis [15]. In clinical practice, response to antiparasitic treatment is often considered a useful diagnostic indicator.

The present study aimed to evaluate the prevalence of Demodex infestation in patients with moderate‐to‐severe AD receiving dupilumab therapy compared with AD patients not receiving systemic immunosuppressive treatment. In addition, we sought to explore the association between Demodex infestation and the occurrence of persistent facial and neck symptoms—such as erythema, pruritus, burning, and scaling—during the course of dupilumab treatment.

## 2 Methods

### 2.1 Study Design and Ethics

This retrospective observational study included patients with a confirmed diagnosis of moderate‐to‐severe AD who were followed at a tertiary dermatology center and evaluated for Demodex infestation between January 2022 and December 2024. Patients in the study group had received dupilumab therapy during the study period. The study protocol was conducted in accordance with the principles of the Declaration of Helsinki. Written informed consent was obtained from all patients for the use of clinical images.

### 2.2 Inclusion Criteria

Patients in the study group were adults with a confirmed diagnosis of moderate‐to‐severe AD who were receiving dupilumab therapy at the time of Demodex assessment. The control group consisted of patients with AD who had neither a prior history of nor ongoing systemic immunosuppressive therapy, including biologics, during the study period.

### 2.3 Exclusion Criteria

The following exclusion criteria were applied to both groups:

- Presence of rosacea or any other concomitant dermatologic condition apart from AD,
- Use of topical corticosteroids or calcineurin inhibitors on the sampled areas within 4 weeks prior to Demodex sampling,
- Demodex assessment not performed using the SSSB technique,
- Incomplete clinical or laboratory data in the electronic medical record.

### 2.4 Data Collection

Sociodemographic characteristics (age and sex) and clinical variables were retrospectively retrieved from electronic medical records. Collected clinical data included disease duration, duration of dupilumab therapy, and the presence or onset of cutaneous symptoms such as facial erythema, pruritus, burning, and scaling. Laboratory parameters included peripheral blood eosinophil counts and total serum immunoglobulin E (IgE) levels, assessed before and after dupilumab initiation when available.

### 2.5 Demodex Evaluation

Demodex assessment was performed during ongoing dupilumab therapy at an index visit; no predefined or fixed time point relative to treatment initiation was established. All patients underwent clinical examination and dermoscopic evaluation. In some cases, dermoscopy revealed features suggestive of Demodex infestation, including dilated follicular openings, protruding gray‐white Demodex tails, follicular plugs, and a mildly erythematous to hyperpigmented background [16].

SSSB samples were obtained from clinically affected areas or sites exhibiting dermoscopic features suggestive of Demodex infestation. When such findings were absent, sampling was performed from standardized facial regions with prominent follicular density, including the lateral nasal or malar areas. Demodex density was quantified using the SSSB method as described by Forton et al. [17]. The number of mites per square centimeter was recorded, and a density of ≥ 5 mites/cm^2^ was considered indicative of positive infestation, in accordance with previously proposed criteria distinguishing significant infestation from simple colonization.

### 2.6 Standardized Skin Surface Biopsy Procedure

For SSSB, a 1 cm^2^ area was delineated on a microscope slide using a waterproof marker. A drop of cyanoacrylate adhesive was applied to the opposite surface of the slide, which was then gently pressed onto the selected skin area for 1 min. After polymerization, the slide was carefully removed, clarified with one to two drops of immersion oil, and covered with a coverslip. Samples were examined under light microscopy at ×10 and ×40 magnifications.

### 2.7 Statistical Analysis

Statistical analyses were performed using SPSS Statistics for Windows, Version 21.0 (IBM Corp., Armonk, NY, USA). Data distribution was assessed using the Shapiro–Wilk test. Continuous variables were expressed as mean ± standard deviation (SD) for normally distributed data and as median with interquartile range for non‐normally distributed data. Categorical variables were presented as counts and percentages.

Comparisons between groups were conducted using the Student's *t*‐test for normally distributed continuous variables, the Mann–Whitney *U* test for non‐normally distributed continuous variables, and the chi‐squared test for categorical variables. To evaluate the potential confounding effect of age on Demodex positivity, binary logistic regression analysis was performed including age and treatment group as independent variables. Odds ratios (ORs) with 95% confidence intervals (CIs) were calculated. All statistical tests were two‐sided, and a *p*‐value < 0.05 was considered statistically significant.

## 3 Results

### 3.1 Study Population

A total of 61 patients were included in the study: 31 patients with moderate‐to‐severe AD receiving dupilumab therapy and 30 patients with AD who were not receiving systemic immunosuppressive treatment. Demographic and baseline clinical characteristics of the study population are summarized in Table 1. There were no statistically significant differences between the dupilumab‐treated group and the control group with respect to age (*p* = 0.250) or sex distribution (*p* = 0.154).

**TABLE 1:** Demographic and clinical characteristics of the study groups.

	Dupilumab‐treated group (*n* = 31)	Control group (*n* = 30)	*p*
Age, years (mean ± SD)	46.68 ± 19.67	52.47 ± 19.20	0.250
Sex, *n* (%)			
Female	14 (45.2)	19 (63.3)	0.154
Male	17 (54.8)	11 (36.7)	
*Demodex* positivity, *n* (%)	14 (45.2)	2 (6.7)	0.001

### 3.2 Comparison of Demodex Positivity Between Groups

**FIGURE 1:**
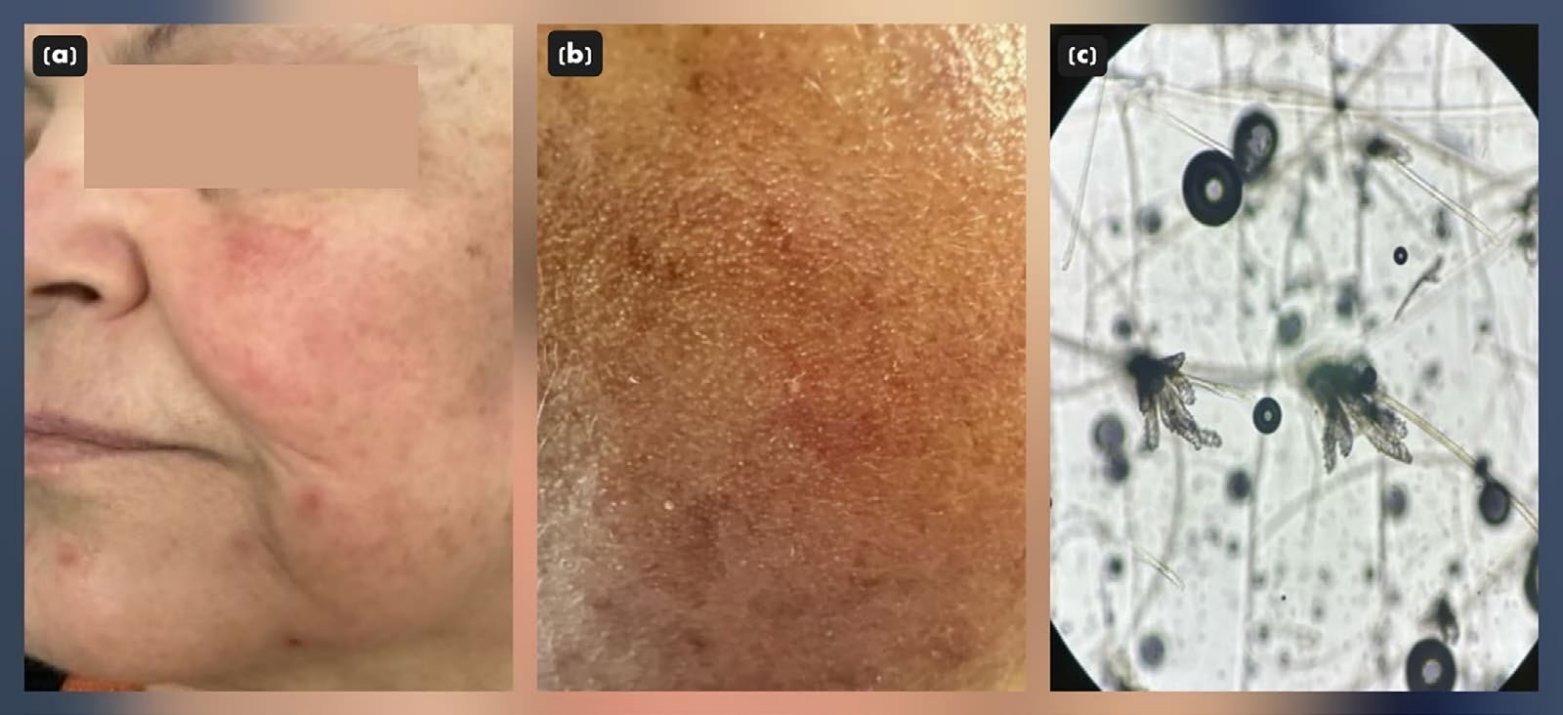
Clinical, dermoscopic, and microscopic features of Demodex infestation. (a) Facial erythema and papulopustular lesions in a 68‐year‐old woman. (b) Dermoscopy (×20) showing dilated follicular openings with protruding gray‐white Demodex tails and follicular plugs. (c) Light microscopy (×40) of standardized skin surface biopsy demonstrating numerous Demodex mites.

Demodex positivity, defined as a SSSB density of ≥ 5 mites/cm^2^, was observed significantly more frequently in patients receiving dupilumab compared with the control group (45.2% vs. 6.7%, respectively; *p* = 0.001). Representative clinical and microscopic findings are shown in Figure 1a–c.

### 3.3 Dupilumab‐Treated Group Subanalysis

Within the dupilumab‐treated cohort (*n* = 31), the duration of dupilumab therapy at the time of Demodex assessment ranged from 3 to 60 months, with a mean duration of 21.16 ± 15.19 months. Sixteen patients (51.6%) had received dupilumab for 3–12 months, whereas 15 patients (48.4%) had been treated for more than 12 months. At the time of Demodex sampling, all patients in the dupilumab‐treated group had achieved at least a 75% improvement in the Eczema Area and Severity Index (EASI) compared with baseline.

Among patients receiving dupilumab, the mean age of Demodex‐positive individuals was significantly higher than that of Demodex‐negative individuals (54.93 ± 17.61 years vs. 39.88 ± 19.09 years, respectively; *p* = 0.031). Demodex positivity was observed in 35.7% of female patients and 52.9% of male patients, with no statistically significant difference between sexes (*p* = 0.337).

Clinical symptoms were reported by 11 of the 14 Demodex‐positive patients receiving dupilumab (78.6%). The most frequently reported symptoms were facial erythema and pruritus, followed by ocular and other cutaneous manifestations (Table 2). In Demodex‐positive patients treated with dupilumab, mite density assessed by SSSB ranged from 5 to 10 mites/cm^2^, with a mean density of 6.86 ± 1.46 mites/cm^2^.

**TABLE 2:** Clinical manifestations in Demodex‐positive patients receiving dupilumab (*n* = 14).

Symptom	*n*	%
Erythema	8	57.1
Pruritus	8	57.1
Conjunctivitis	7	50.0
Blepharitis	5	35.7
Papulopustular lesions	3	21.4
Scalp involvement	2	14.3
Post‐treatment acneiform eruptions	2	14.3

Following dupilumab therapy, the mean total serum immunoglobulin E (IgE) level was 4727.36 ± 10126.68 IU/mL in the Demodex‐positive group and 1878.71 ± 1753.17 IU/mL in the Demodex‐negative group; this difference was not statistically significant (*p* = 0.450, Mann–Whitney *U* test). In the Demodex‐positive group, mean peripheral eosinophil counts decreased from 508.57 ± 616.42/μL before dupilumab therapy to 417.14 ± 405.49/μL after therapy. Corresponding values in the Demodex‐negative group were 443.53 ± 315.04/μL and 354.71 ± 311.35/μL, respectively. No statistically significant differences in eosinophil counts were observed between Demodex‐positive and Demodex‐negative patients either before (*p* = 0.316) or after dupilumab treatment (*p* = 0.984).

In an additional subgroup analysis, Demodex‐positive patients receiving dupilumab were further stratified according to the presence of clinical symptoms (symptomatic vs. asymptomatic). No statistically significant differences were observed between symptomatic and asymptomatic Demodex‐positive patients with respect to age, serum IgE levels, pre‐treatment eosinophil counts, duration of dupilumab treatment, or mite density assessed by SSSB (all *p* > 0.05).

### 3.4 Multivariate Analysis of Factors Associated With Demodex Positivity

To evaluate the potential confounding effect of age on Demodex positivity, binary logistic regression analysis was performed including age and treatment group as independent variables. Increasing age was independently associated with Demodex positivity (OR = 1.049, 95% CI: 1.009–1.090, *p* = 0.016). However, after adjustment for age, dupilumab treatment remained significantly associated with Demodex positivity (OR = 20.518, 95% CI: 3.385–124.355, *p* = 0.001).

## 4 Discussion

In this study, Demodex positivity was significantly more frequent in patients with moderate‐to‐severe AD receiving dupilumab therapy compared with an AD control group with similar age and sex distribution (SSSB ≥ 5 mites/cm^2^: 45.2% vs. 6.7%; *p* = 0.001). Although patients in the dupilumab‐treated group initially fulfilled criteria for moderate‐to‐severe AD based on treatment indication, disease severity was mild and comparable at the time of Demodex assessment, as all patients had achieved at least an EASI‐75 response. The absence of Demodex‐related or rosacea‐like complaints prior to treatment in both groups, together with the persistence of facial and ocular symptoms despite topical therapies in the dupilumab‐treated group, may suggest a temporal association between dupilumab treatment and the observed clinical findings. However, given the retrospective design and lack of baseline Demodex assessment, a causal relationship cannot be established. In addition, asymptomatic pre‐existing Demodex colonization cannot be excluded, as microscopic evaluation was not routinely performed before dupilumab initiation in patients without facial or ocular symptoms. Therefore, our findings should be interpreted as an association rather than evidence of a direct causal relationship.

The higher mean age observed among Demodex‐positive patients compared with Demodex‐negative patients (54.93 ± 17.61 years vs. 39.88 ± 19.09 years; *p* = 0.031) further indicates that host‐related factors may contribute to Demodex positivity. The use of quantitative Demodex assessment by SSSB minimizes the likelihood of detecting incidental colonization and strengthens the reliability of the observed intergroup difference.

Dupilumab exerts its therapeutic effect by inhibiting IL‐4 and IL‐13 signaling through blockade of the IL‐4 receptor alpha subunit, thereby suppressing Th2‐mediated inflammation [18]. Th2 immune responses play a role in host defense against parasitic organisms, and modulation of this pathway may alter the cutaneous immune environment [8]. It has been proposed that suppression of Th2 signaling may lead to a relative shift toward Th1/Th17 immune responses [7, 19]. In this context, Kim et al. reported increased IL‐17 levels in tear samples of patients with Demodex infestation [20]. Although these observations do not establish causality, they suggest that immune pathway alterations may be relevant to Demodex proliferation or persistence under certain clinical conditions. Accordingly, the increased frequency of Demodex positivity observed in our dupilumab‐treated cohort may reflect changes in the local immune milieu rather than a direct treatment‐related adverse effect.

No significant differences in total IgE levels or peripheral eosinophil counts were observed between Demodex‐positive and Demodex‐negative patients, suggesting that Demodex positivity was not associated with these systemic biomarkers in our cohort.

Several mechanisms have been proposed to explain persistent head‐and‐neck erythema and associated symptoms during dupilumab therapy, including exacerbation of allergic contact dermatitis, hypersensitivity reactions, psoriasiform inflammation, and seborrheic dermatitis–like reactions potentially associated with Demodex or Malassezia species in facial regions˒ [7, 21]. Real‐world studies have reported head‐and‐neck involvement in up to approximately 10% of patients receiving dupilumab [21]. In line with previous reports, the predominance of erythema, pruritus, conjunctivitis, blepharitis, and papulopustular lesions localized to the head‐and‐neck region and showing limited response to topical anti‐inflammatory therapies highlights the importance of considering alternative or coexisting diagnoses. In such cases, evaluation for Demodex infestation may be clinically relevant, particularly in patients with persistent or atypical facial and ocular symptoms [7, 18, 19, 21].

Furthermore, subgroup analysis of Demodex‐positive patients receiving dupilumab did not demonstrate significant differences between symptomatic and asymptomatic individuals in terms of age, treatment duration, serum IgE levels, eosinophil counts, or mite density. These findings suggest that Demodex positivity alone may not be sufficient to determine the development of clinically apparent manifestations and support the possibility that additional host‐related or immunological factors may contribute to symptom expression.

An important consideration is the potential influence of age on Demodex colonization, since previous studies have consistently demonstrated increasing Demodex prevalence with advancing age. In our study, additional regression analysis confirmed that increasing age was independently associated with Demodex positivity. However, the control and dupilumab‐treated groups did not significantly differ in age distribution, and the association between dupilumab treatment and Demodex positivity remained significant after adjustment for age. Therefore, although age may contribute to Demodex positivity, it is unlikely to fully explain the observed association.

Current evidence regarding the relationship between Demodex infestation and dupilumab therapy remains limited, with most published data consisting of case reports and small case series [7, 22]. In these reports, findings obtained through dermoscopy, confocal microscopy, and histopathology have been partly consistent with demodicosis [18, 22, 23]. By incorporating a control group and quantitative Demodex assessment, the present study adds controlled real‐world data to the existing literature and supports an association between dupilumab treatment and increased Demodex positivity. Recognition of potential Demodex‐related manifestations may also be clinically relevant, as appropriate evaluation and targeted treatment could help avoid unnecessary modification or discontinuation of an otherwise effective therapy. Nevertheless, prospective studies with baseline and longitudinal Demodex assessments are required to clarify the temporal relationship and underlying mechanisms.

## 5 Limitations

This study has several limitations. Its single‐center and retrospective design limits generalizability and does not allow causal inferences. In addition, disease severity was not fully matched between the dupilumab‐treated and control groups, as controls were not receiving systemic immunosuppressive therapy; therefore, residual confounding related to underlying disease characteristics cannot be excluded despite comparable age and sex distributions.

Demodex assessment was performed at a single time point during dupilumab therapy, and baseline Demodex density prior to treatment initiation was unavailable, precluding evaluation of temporal changes in colonization. Furthermore, microscopic screening was not routinely performed in asymptomatic patients before dupilumab initiation; therefore, pre‐existing asymptomatic colonization cannot be excluded. Although additional analyses were performed to address potential confounding factors, residual confounding cannot be entirely excluded because of the observational design and relatively small sample size. Prospective longitudinal studies are warranted to clarify these findings.

## 6 Conclusion

In conclusion, erythema and associated symptoms involving the head‐and‐neck region were more frequently observed in atopic dermatitis patients receiving dupilumab therapy who were also found to have Demodex infestation. Although a causal relationship cannot be established, these findings suggest that Demodex infestation should be considered in dupilumab‐treated patients presenting with persistent or atypical facial and ocular symptoms. Recognition of possible coexisting Demodex‐related manifestations may be clinically relevant, as appropriate evaluation and targeted management could potentially help avoid unnecessary modification or premature discontinuation of an otherwise effective therapy. Further prospective studies with baseline and longitudinal assessments are needed to clarify the temporal relationship and underlying mechanisms.

## Funding

The authors have nothing to report.

## Ethics Statement

The study was approved by the local Clinical Research Ethics Committee (Approval No: 854/2025) and conducted in accordance with the Declaration of Helsinki.

## Consent

Written informed consent was obtained from all participants for the SSSB.

## Conflicts of Interest

The authors declare no conflicts of interest.
